# Supplementary material for: Importance of feeding status evaluation in older patients undergoing hemodialysis
Source: PLoS One. 2023 Jan 3;18(1):e0279199. doi: 10.1371/journal.pone.0279199 (PMC9810173; doi:10.1371/journal.pone.0279199)
Supplement: S1 Table — Wilcoxon sum rank test was used. (DOCX) [file pone.0279199.s002.docx]

**Supplementary Table 1. Association between major items of the Kuchikara Taberu Balance Chart and Dementia**

|  | overall condition | cognitive function  while eating | severity of pharyngeal  dysphagia | position and endurance  while eating | food  modification | nutrition |
| --- | --- | --- | --- | --- | --- | --- |
| Dementia (+) | 3.8 ± 1.2 | 4.2 ± 1.0 | 4.3 ± 1.0 | 3.8 ± 1.0 | 3.3 ± 1.2 | 2.9 ± 1.6 |
| Dementia (-) | 4.0 ± 1.3 | 4.7 ± 0.8 | 4.9 ± 0.4 | 4.2 ± 1.1 | 4.4 ± 1.2 | 3.3 ± 1.5 |
| *p* | 0.49 | 0.01 | <0.001 | 0.01 | <0.001 | 0.23 |
