# Supplementary material for: Importance of feeding status evaluation in older patients undergoing hemodialysis
Source: PLoS One. 2023 Jan 3;18(1):e0279199. doi: 10.1371/journal.pone.0279199 (PMC9810173; doi:10.1371/journal.pone.0279199)
Supplement: S2 Table — Spearman’s rank correlation coefficient was used. (DOCX) [file pone.0279199.s003.docx]

**Supplementary Table 2. Association between major items of the Kuchikara Taberu Balance Chart and nutritional indicators**

|  | overall condition | | cognitive function  while eating | | severity of pharyngeal  dysphagia | | position and endurance  while eating | | food  modification | | nutrition | |
| --- | --- | --- | --- | --- | --- | --- | --- | --- | --- | --- | --- | --- |
|  | ρ | *p* | ρ | *p* | ρ | *p* | ρ | *p* | ρ | *p* | ρ | *p* |
| Serum albumin level | 0.368 | 0.001 | 0.359 | 0.001 | 0.335 | 0.002 | 0.391 | <0.001 | 0.476 | <0.001 | 0.201 | 0.07 |
| Geriatric Nutritional Risk Index | 0.265 | 0.02 | 0.443 | <0.001 | 0.438 | <0.001 | 0.430 | <0.001 | 0.551 | <0.001 | 0.437 | <0.001 |
| Nutritional Risk Index | - 0.253 | 0.02 | -0.392 | <0.001 | -0.391 | <0.001 | -0.303 | 0.006 | -0.486 | <0.001 | -0.495 | <0.001 |
